# Automated Bone Marrow Cell Classification for Haematological Disease Diagnosis Using Siamese Neural Network

**DOI:** 10.3390/diagnostics13010112

**Published:** 2022-12-29

**Authors:** Balasundaram Ananthakrishnan, Ayesha Shaik, Shivam Akhouri, Paras Garg, Vaibhav Gadag, Muthu Subash Kavitha

**Affiliations:** 1 Centre for Cyber Physical Systems, Vellore Institute of Technology, Chennai 600127, India; 2 School of Computer Science and Engineering, Vellore Institute of Technology, Chennai 600127, India; 3 School of Information and Data Sciences, Nagasaki University, Nagasaki 8528521, Japan

**Keywords:** Siamese network, deep learning, bone marrow, contrastive loss function, image convolution

## Abstract

The critical structure and nature of different bone marrow cells which form a base in the diagnosis of haematological ailments requires a high-grade classification which is a very prolonged approach and accounts for human error if performed manually, even by field experts. Therefore, the aim of this research is to automate the process to study and accurately classify the structure of bone marrow cells which will help in the diagnosis of haematological ailments at a much faster and better rate. Various machine learning algorithms and models, such as CNN + SVM, CNN + XGB Boost and Siamese network, were trained and tested across a dataset of 170,000 expert-annotated cell images from 945 patients’ bone marrow smears with haematological disorders. The metrics used for evaluation of this research are accuracy of model, precision and recall of all the different classes of cells. Based on these performance metrics the CNN + SVM, CNN + XGB, resulted in 32%, 28% accuracy, respectively, and therefore these models were discarded. Siamese neural resulted in 91% accuracy and 84% validation accuracy. Moreover, the weighted average recall values of the Siamese neural network were 92% for training and 91% for validation. Hence, the final results are based on Siamese neural network model as it was outperforming all the other algorithms used in this research.

## 1. Introduction

The use of optical microscopes to evaluate and categorize samples of bone marrow cells is a century-old practice for diagnosing blood diseases. The strategy of searching for uncommon but diagnostically essential cells is well-established, but time-consuming and difficult. Highly skilled individual with long term experience is a necessity [1]. However, the conclusion of those findings is subjective, and there is no statistical standard in place yet. The increased density of contacting and overlapping cells, as well as the greater diversity and complexity of cell morphologies, make detection and categorization in BMA smears substantially more difficult. Without good cell identification and localisation of cell borders, classification is challenging, and the minor distinctions in cytologic properties required to differentiate various cell types found in bone marrow make it even more difficult. [2]. In the diagnosis of malignant and non-malignant illnesses of the hematopoietic system, the examination and distinction of bone marrow cell morphologies are critical [3].

**Figure 1:**
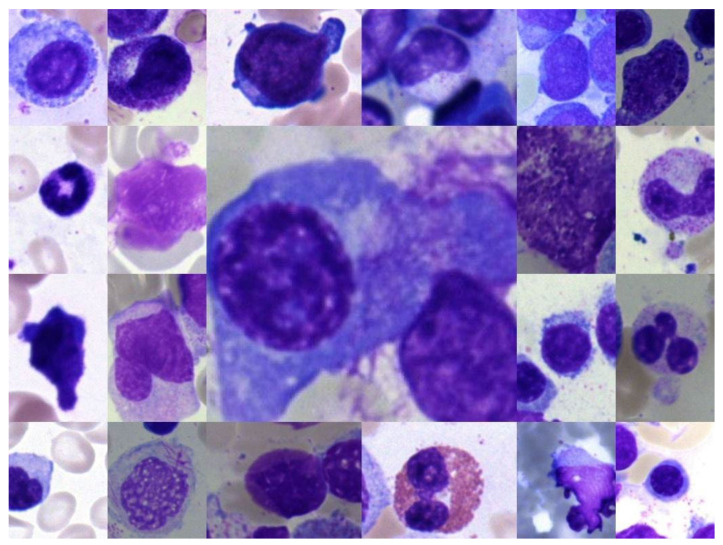
Bone marrow cells of 21 different classes.

**Figure 2:**
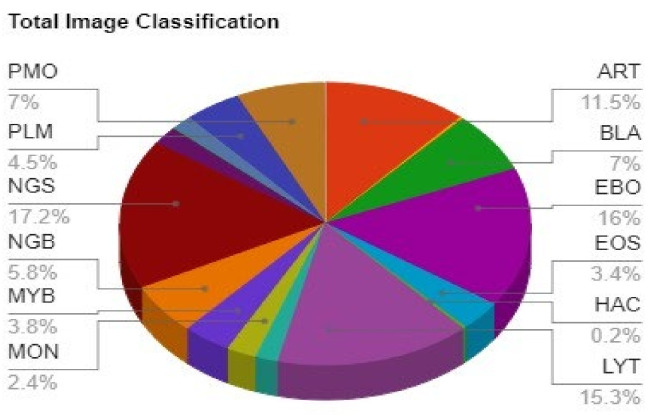
Pie chart depicting the fraction of images for each class out of 170,000 images.

Previous studies have been applied to the bone marrow problem, but they were all predicated on the premise that manually segmented pictures were accessible. Machine Learning techniques adopted for this purpose were mainly focused on cherry picking a particular subset of classes consisting of more data points, which may fail to classify those excluded data points [4,5] Other models which took the whole dataset in consideration were able to achieve state of the art precision in classes consisting of a large number of datapoints, but failed to achieve such figures in those classes which are deficient of enough data points [6,7,8]. Furthermore, most prior investigations of automated cytomorphologic identification concentrated on the classification of physiological cell types or peripheral blood smears as shown in Figure 1, restricting their applicability to the characterization of leukocytes in the BM for haematological malignancies diagnosis. Recent advancements in this field have focused mainly upon applying efficient Image segmentation and classification techniques; however, class imbalance as presented in Figure 2 played a major role in the classification task to achieve overall accuracy [9,10].

Bone marrow is a dynamic tissue compartment in the cavity of bones. In adults, haematopoietic cells are produced by the bone marrow cells in the large bones that account for 2–5% of an adult’s weight. Bone marrow imaging is a diagnostic tool for determination and staging of several haematological bone marrow disorders [3]. The sample acquisition process is mainly accomplished using either bone marrow aspiration or bone marrow biopsy techniques. These examinations are quite useful in finding out the root cause of problem with RBC’s (red blood cells), WBC’s (white blood cells) and platelets as well as monitoring related diseases such as polycythaemia vera, thrombocytopenia and anaemia including certain type of cancers such as lymphoma, leukaemia and multiple myeloma [1].

Because of the uneven staining and high-cell population, segmenting the nucleus and cytoplasm of leukocytes from bone marrow pictures is a complex procedure. The nucleus and cytoplasm of leukocytes were extracted using a combination of segmentation techniques such as thresholding, edge detection, pixel clustering and area growth. These approaches have been used since the photographs had homogeneous backgrounds and strong contrast, allowing the things of interest to be clearly distinguished. Samples generated for daily investigations in haematology laboratories, on the other hand, are frequently not consistent [6]. Few studies integrate training techniques such as SVM, mean-shift and coloured image segmentation to provide an automated AI method for finding models of various regions of an image. When compared to the thresholding and watershed algorithms, these approaches yield greater segmentation accuracy in complicated situations and are more resistant to colour confusion and alterations. Few strategies have been proposed to tackle the problem of overlaying blood cells. The underlying algorithm separates the cells either by employing dividing lines to combine concave points or by applying thresholding techniques and expanding the ROI while keeping the form [7].

Diagnosis of bone marrow is a time-consuming process as well as it requires proper infrastructure of diagnosis tools to spin up the process. Since it involves use of complex methodology and tools, the bearing cost is also quite high. Deep learning classification models currently available with high accuracy are not trained with all the classes and those which have been trained with all the classes fail to achieve acceptable accuracy to be utilised for medical diagnosis. This paper presents a way to fill this trade off gap by adopting a model that achieves high accuracy even with low density classes.

In our work, we have focused on improving the overall accuracy of classification. The dataset consists of more than 170,000 bone marrow images with very acute class imbalance. Thus, the main motivation was to find a way to eradicate the effect of this imbalance to train a deep neural network that has very high overall accuracy. The key tool that we adopted to counter the problem of class imbalance is the use of the Siamese network. The main key feature of this network is that it works well even when we are in shortage of enough data points, which is exactly the issue we are facing with some of the data classes. While testing the Siamese model, it outperformed XGB and SVM model with a training accuracy more than 91% and valid accuracy of over 84%. The model also performed well with individual class classification.

The remainder of the paper is laid out as follows: Section 2 contains a literature survey in the area of bone marrow morphology. Section 3 contains a proposed system and algorithms. Section 4 proposes the experimental results, discussion and performance analysis. The study is concluded in Section 5, with future directions.

## 2. Literature Review

Bone marrow cell classification is a key area in the prognosis of diseases related to blood. Many classification models have been proposed related to transfer learning CNN models, DC-GAN with ResNet combination and VGG based implementation for cell classification. In the following paragraphs we present an overview of those methods.

Study [8] proposed a model that combined DC-GAN with ResNet for classification of blood cells and adopted a transfer learning approach on ImageNet dataset. The results shows that there is an increase in accuracy by 1.2% on DC-GAN enhanced images with an overall test accuracy of 91.68%. This research [11] adopted a CNN based approach consisting of VGG16 and InceptionV3 to classify blood cell type against 17,902 digital images and eight classes. The overall accuracy was 90%; however, there was a large variation in true positives rates for individual classes in both the approaches. Another study [5] proposed a rapid localization of bone marrow and self-generating ROI with a patch-based deep learning model for the classification of 16 kinds of cell. The proposed model was trained on 12,426 annotated cells with an overall validation recall of more than 90.5%. The results of a test conducted on another batch of 3000 images, achieving accuracy of more than 98%, show that the model does not overfit the training data at all.

Currently, due to the exploding development in deep learning, increasing research has been conducted on the classification of multiple myeloma. The authors of [9] explored the effectiveness of detection algorithms such as RCNN and detection of multiple myeloma using unet, for which they used 85 high resolution microscopic images. The model possessed a state-of-the-art training loss of 0.05 after 400 epochs, but with a magnificent difference in validation loss. Despite the fact that Mask-RCNN is capable of addressing most of the issues associated with myeloma cell segmentation, it failed to detect some of the under-stained cells when the colour contrast between cytoplasm and background is lower.

At the same time, DL systems for the concurrent detection and classification in histology image analysis deal with the location and distinct types of nuclei/cells. Traditionally, these two challenges have been tackled separately, necessitating more training time. To solve this time constraint issue, this research [10] came up with a concatenated asymmetric DL structure (Syn-AHDA) for efficiently accounting of pictures with deformed features and noise effects. The experimental dataset includes 10,496 annotated images. The proposed model achieved detection precision of 92.66% and classification precision of 87.12%. The main feature of the Syn-ADHA network is that it produced competitive accuracy but took almost two-thirds of the overall training time as compared to another network.

The studies [12,13,14] created an AI system that evaluates blood cell dysplasia on bone marrow smears as well as displays the outcome of AI forecast for one of the dysplasias that is most representative: reduced granules. The classifier’s performance was assessed using a five-fold cross-validation after the detector, which was built on a deep learning model pre-trained with natural pictures and resulted in 97.2% and 98.2% accuracy, respectively, when labels for DG1–3 are deemed positive and DG1 is not used. In order to do a differential cell count on bone marrow smears to aid in diagnosis, the authors of [15] built an autonomous system and pathologists using microscopes conducted the study in parallel with machine automatic recognition and traditional manual differential count. The result of classification was based on specificity, sensitivity and accuracy and the effectiveness of the approach in classifying the cells in BM smears was validated with an overall validation accuracy of 90.1%. It may be able to help with the clinical application of BM smear examination.

The purpose of this [16] study is to determine whether transcriptomic approach machine learning can predict the presence of AML without expert input. They took into account a variety of real-world situations, such as cross-study problems and prediction across several technology platforms, and discovered that reliable prediction is feasible in a variety of situations and, in many instances, with only a small number of training samples. However, they also demonstrated that, based on the instance and the corresponding prevalence, huge training sets might be necessary to achieve accuracies good enough to produce respectable PPVs. The findings indicate that it may be possible to attain decent performance in a nearly automated manner with current technologies. This [17] study focuses on the part that senescence plays in the bone marrow (BM) microenvironment’s ageing process. Numerous BM conditions are age-related illnesses that heavily rely on the bone marrow microenvironment. The study resulted in 97.6% accuracy in classifying AML bone marrow type v/s other types in dataset 1 comprising 2500 samples, 98% accuracy in 8348 samples from Affymetrix HG U133 2.0 Micro array and 99.1% accuracy from 1181 samples obtained using RNAseq.

This [18] study created a machine learning model for MDS prediction one year before the condition was clinically diagnosed. A total number of 790,470 patients participated in this trial, of whom 1428 received an MDS diagnosis in the end, whereas 789,042 did not. The XGB model was contrasted with two additional models: artificial neural networks and logistic regression, two alternative machine learning methods. This [19] study showed that cancer/MSC cell fusion, a relatively uncommon occurrence, can impart chemotherapy resistance in addition to other PR tumorigenic features to malignancies that have been known to attract MSC. They examined the association between MSC fusion and the pattern of gene expression in SCC-25 cancer cells. Other noteworthy findings were that only 21% of those in the negative class test set had previously been diagnosed with cancer, compared to 33% of those in the positive class set.

Although classifying the blood cells manually is frequently employed in clinics and hospitals, it is not time-effective and has an impact on clinical workflow. Automated cell classification technologies could help clinicians make diagnoses more quickly and accurately. Convolutional Neural Network and Support Vector Machine (CNN-SVM) was presented in this [20] study as a solution to this issue, with Convolutional Neural Network model being utilised to directly extract features from the pictures. Neutrophil, lymphocyte, monocyte, eosinophil and pathogenic white blood cells are the five categories of white blood cells that may be classified using the features that were collected. On 15,764 enhanced pictures, three distinct CNN models, including Alexnet, Resnet-101 and VGG-19, were tested to determine which one would be the most effective at extracting features. Resnet-101 was chosen to categorise five categories of white blood cells because it had the highest accuracy rate of (97.8%) when combined with SVM. Examining the bone marrow (BM) is a crucial first step in the diagnosis and treatment of many hematologic diseases. For the purpose of BM NDC WSI analysis, this study [9] presented an effective and entirely autonomous hierarchical deep learning system. The suggested hierarchical framework is made up of three parts: (1) a deep learning model for quick localization of BM particles and cellular trails that creates regions of interest (ROI) for additional analysis; (2) a patch-based deep learning approach for cell recognition of 16 cell types, that have not been shown in prior studies; and (3) a fast-stitching model for combining patch-based results. The suggested method outperforms the current benchmark small-image-based techniques in terms of recall, accuracy and computing efficiency. Table 1 lists the summary of finding from other papers.

## 3. Material and Methods

In this paper, we proposed a probabilistic Siamese network with triplet loss function for bone marrow cell classification. For justified comparison, 2 parallel models are trained against identical training, test and validation which include:

CNN + SVM

CNN + Xgboost

The Bone Marrow Cell Classification dataset available in Kaggle [21] was used for this work. The dataset comprises more than 170,000 annotated images taken from the bone marrow smears of 945 patients using the May-Grünwald-Giemsa/Pappenheim stain [22]. The images had been acquired using a brightfield microscope with 40x magnification and oil immersion. The samples had been processed in the Munich Leukaemia Laboratory (MLL), scanned using equipment developed at Fraunhofer IIS and post-processed using software developed at Helmholtz Munic [23].

**Figure 3:**
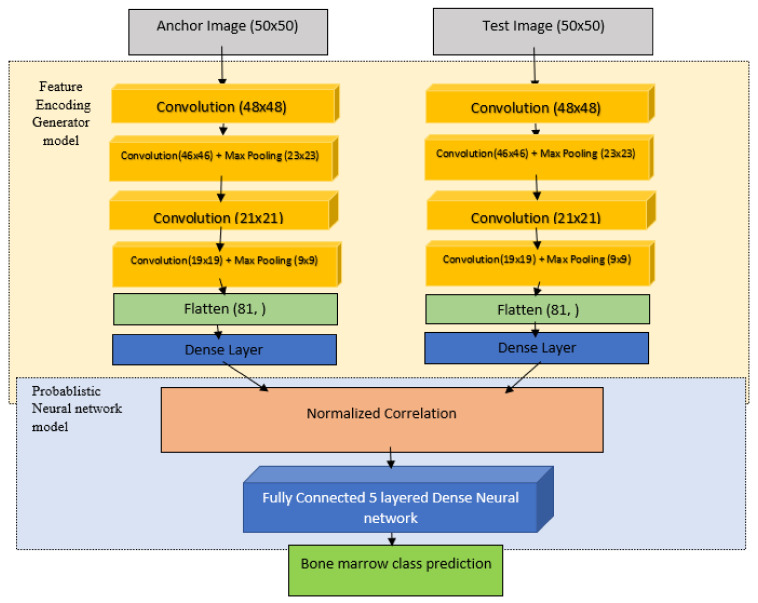
Block diagram of proposed Siamese neural network model.

The block diagram for the model is present in Figure 3. The proposed model is composed of:

Four convolution layers of paired input images accompanied by Max Pooling layer. A concatenation layer to feed forward the output of both the convolution + dense transforms. Five Dense layers to predict whether the provided paired images were of same class or not.

This methodology is quite effective in mitigating the vast accuracy variation due to imbalance in the dataset classes. In short, Siamese network learns differentiating features among other networks in a must faster rate as compared to other persisting models.

### 3.1. Siamese Network

Siamese network, often called twin neural network proposed by Chicco, Davide, which uses the identical training parameters for both the given inputs for accomplishing the classification task. Out of the given pair, one of the inputs has already been assigned with a particular label, thus limiting the job of the network to extract identical or differentiating features out of the pair.

For learning the parameters, the Siamese model applies an optimization function on the triplet loss function. Triplet loss function requires one anchor data point as well as one classification data point with either a positive or negative label. If the data point is positive, it means that both the fed inputs belong to the same class, and vice versa. The model includes two series of networks, one for generating the encodings out of the image, and the other solemnly dedicated to the classification task from those acquired encodings. The primary objective of triplet loss function is the difference between anchor and positive data point to be smaller than anchor and negative data point, i.e.,

(1) $$\left|\left|f\left(anchor\right)-f\left(positive\right)\right|\right|<\left|\left|f\left(anchor\right)-f\left(negative\right)\right|\right|+\varepsilon$$

where,

*f(n) is encodings of input data points*

ε often known as margin to nullify the trivial solution.

Thus, the overall triplet loss function is defined as:

(2) $$L\left(anchor,\;positive,\;negative\right)=max\left(\left|\left|f\left(anchor\right)-f\left(positive\right)\right|\right|-\left|\left|f\left(anchor\right)-f\left(negative\right)+\varepsilon \right|\right|,\;0\right)$$

The max function is applied intentionally, so that the loss function does not backfires itself when we obtain the desired negative loss, that is, when the overall loss

(3) $$\mathrm{Loss}=\Sigma \;L\left(\mathrm{anchor}\left(i\right),\;\mathrm{positive}\left(i\right),\;\mathrm{negative}\left(i\right)\right)$$

is positive the model will try to improve the parameters using the optimising algorithm provided to the model, and when the first argument to the max function turns out to be negative, it will take zero instead, so that loss function for that particular pair does not have any effect on the optimising algorithm.

The dataset consists of paired 50 × 50 images and corresponding similarity labels. We try to maximise this similarity label prediction through hidden representation generated by the encoding layer of the input. The conditional likelihood is given by:

(4) $$P\left(h1,\;h2,\;\varepsilon \right)=\prod (g{\left(h1,\;h2,\;\epsilon \right)}^{s}*\;{\left[1-g\left(h1,\;h2,\;\varepsilon \right)\right]}^{1-s})$$

where,

*h1* = encoding generated by anchor image,

*h2* = encoding generated by test image

*g*(*h1*, *h2*, *e*) = contrastive loss function

*s* = similarity label

ε = margin

After taking natural log both side in (1):

(5) $$L\left(s\;|\;h1,\;h2,\;\varepsilon \right)=\sum (\left(s\right)\left(log\;\left(g\left(h1,\;h2,\;\varepsilon \right)\right)+\left(1-s\right)\left[log\;\left(1-g\left(h1,\;h2,\;\varepsilon \right)\right)\;\right]\right)\;$$

where, *L* = Contrastive loss function

The hidden encodings are generated using forward passing the image to the convolution layer followed by a flatten and dense layer with ReLU activation. In Siamese network, the model parameters, as well as the network parameters, are simultaneously optimised by taking the log likelihood w.r.t. similarity model given by:

(6) $$\frac{\partial L}{\partial \alpha }=\sum \frac{S^{m}-g^{m}}{g^{m}\left(1-g^{m}\right)}*\;\frac{\partial g\left(h1^{m},h2^{m},\;\alpha \right)\;}{\partial \alpha }$$

(7) $$\frac{\partial L}{\partial h1}=\sum \frac{S^{m}-g^{m}}{g^{m}\left(1-g^{m}\right)}*\;\frac{\partial g\left(h1^{m},h2^{m},\;\alpha \right)\;}{\partial h1}$$

(8) $$\frac{\partial L}{\partial h2}=\sum \frac{S^{m}-g^{m}}{g^{m}\left(1-g^{m}\right)}*\;\frac{\partial g\left(h1^{m},h2^{m},\;\alpha \right)\;}{\partial h2}$$

### 3.2. Algorithm

First from the dataset of 170,000 images paired data are generated and then divided into mini batches. Stranding of similar and dissimilar pairs are kept equal for each of the class labels. The pairs are then passed through a model where the network parameters and conditional probability parameters are jointly updated.

**Create** training pair images

**For** n number of epochs

   **For** each image pair do

    **Forward** Propagation

    **Compute** f(anchor) and f(test)

    **Compute** h1^k^ and h2^k^ and g^k^

    **Backward propagation** computing derivatives for both network as well as conditional parameters

   **End for**

   **Update** parameters using adam optimizer function

End for

#### 3.2.1. Forward Propagation

##### Convolution Layer

**Figure 4:**
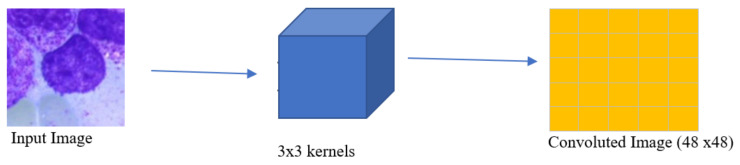
Outline of operation principle of convolution layer with 3 × 3 kernel.

The input image is passed through a series of convolution units as shown in Figure 4 to extract key features from the image to generate optimal encoding for it. Pixel values in convoluted images are generated as the average weighted sum of kernel and neighbouring pixel values. The selection of the desired kernel has also been incorporated as a part of model training itself.

##### Max Pooling Layer

**Figure 5:**
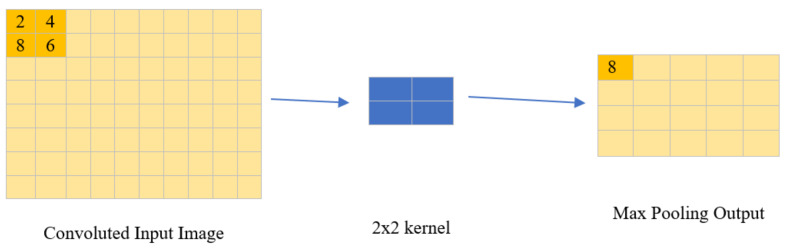
Illustration of function performed by kernel in max pooling layer for each 2 × 2 grid in a convolved image.

After series of convolution the model is passed through max pooling layer where input pixels are taken in a 2 × 2 grid manner and maximum out of it is retained in the output discarding the other 3-pixel values. After each max pooling layer, the dimension of output image becomes the half of input image as demonstrated in Figure 5.

##### Flatten Layer

**Figure 6:**
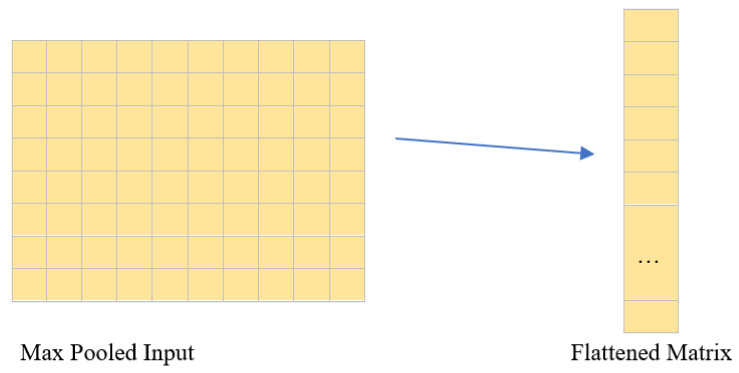
Graphic demonstrating the role of the Flatten layer in converting a 2D grid to a 1D vector.

This layer changes the input 2D matrix to a 1D vector which can be then fed to the Dense layer as shown in Figure 6. The stacking of pixel layers in 1D channel is performed in a row-wise manner.

##### Dense Layer

It is a deeply connected layer of neurons that takes upon the input and generates the result from the following mathematical formula:

(9) $$Output=Activation\left(dot\left(Weight,\;input\right)+bais\right)$$

wherein Weight and bais are model trainable parameters and ReLu activation has been used to introduce non-linearity in the model.

#### 3.2.2. Backward Propagation

The Siamese network learns by back propagating the derivatives according to the chain rule as follows:

For the last Dense layer, derivatives are calculated as:

(10) $$\frac{\partial L}{\partial W^{\left[5\right]}}=\frac{\partial L}{\partial h1}\;\frac{\partial h1}{\partial W^{\left[5\right]}}+\frac{\partial L}{\partial h2}\;\frac{\partial h2}{\partial W^{\left[5\right]}}$$

where *∂h*1*/∂W* and *∂h*2*/∂W* is given by:

(11) $$\frac{\partial h1}{\partial W^{\left[5\right]}}=\frac{\partial h1}{\partial Z1}\frac{\partial Z1}{\partial W^{\left[5\right]}}$$

(12) $$\frac{\partial h2}{\partial W^{\left[5\right]}}=\frac{\partial h2}{\partial Z2}\frac{\partial Z2}{\partial W^{\left[5\right]}}$$

From this the overall set of back propagating derivatives can be derived as follows:

(13) $$\delta 1^{\left[5\right]}=\frac{\partial L}{\partial h1}f\prime \left(Z1^{\left[5\right]}\right)$$

(14) $$\delta 2^{\left[5\right]}=\frac{\partial L}{\partial h2}f\prime \left(Z2^{\left[5\right]}\right)$$

while for layers *l* = 1 to 4 is given as:

(15) $$\delta 1\left[L\right]=\left(W\left[L\right]\;\delta 1\left[L+1\right]\;\right)f\prime \left(z1\left[L\right]\right)$$

(16) $$\delta 2\left[L\right]=\left(W\left[L\right]\;\delta 2\left[L+1\right]\;\right)f\prime \left(z2\left[L\right]\right)$$

#### 3.2.3. Update

The parameters are then updated as follows in the adam optimizer:

$$W^{\left[L\right]}=W^{\left[L\right]}-\eta \left(A_{1}^{\left[L\right]}\;\frac{V_{\delta 1}^{Corrected}}{\sqrt{S_{\delta 1}^{Corrected}+\epsilon }}+A_{2}^{\left[L\right]}\;\frac{V_{\delta 2}^{Corrected}}{\sqrt{S_{\delta 2}^{Corrected}+\varepsilon }}\right)$$

(17) $$b\left[L\right]=b\left[L\right]-\eta \left(\delta 1\left[L+1\right]+\delta 2\left[L+1\right]\right)$$

where *S_δi_* is given by,

(18) $$V_{\delta i}^{corrected}=\frac{V_{\delta i}}{1-\beta 1}$$

(19) $$S_{\delta i}^{corrected}=\;\frac{S_{\delta i}}{1-\beta 2}$$

while *S_δi_* and *V_δi_* equals,

(20) $$V_{\delta i}^{\left[epoch\right]}=V_{\delta i}^{\left[epoch-1\right]}\;\beta 1+\left(1-\beta 1\right)\;\delta i$$

(21) $$S_{\delta i}^{\left[epoch\right]}=S_{\delta i}^{\left[epoch-1\right]}\;\beta 2+\left(1-\beta 2\right)\;\left(\delta i*\;\delta i\right)$$

where *η*, *β*_1_, *β*_2_ are adjustable hyperparameters of the model and initially

*V*_*δ*1_[0] = 0

*V*_*δ*2_[0] = 0

*S*_*δ*1_[0] = 0

*S*_*δ*2_[0] = 0.

## 4. Experimental Results

The dataset used for this work comprises over 170,000 images of expert-annotated cells from the bone marrow smears of 945 patients stained using the May–Grünwald–Giemsa/Pappenheim stain. The images of the dataset were acquired using brightfield microscope with 40x magnification and oil immersion. The dataset comprises 21 unique class labels. The details of the class labels are provided in Table 2.

The development of data-driven, computational approaches in diagnostic medicine requires large datasets with high quality data capture and annotation. Only a few datasets are now publicly accessible for bone marrow cells morphology, a crucial diagnostic technique for a wide range of hematologic disorders. All samples were processed at the Munich Leukaemia Laboratory (MLL), which also used Fraunhofer IIS equipment for scanning and Helmholtz Munich software for post-processing.

The Metrics taken for evaluation of the models are-

(22) $$\mathrm{Accuracy}=\frac{TP+TN}{TP+TN+FN+FP}$$

(23) $$\mathrm{Precision}=\frac{TP}{TP+FP}$$

(24) $$\mathrm{Rec}\;(\mathrm{or}\;\mathrm{Sensitivity})=\frac{TP}{TP+FN}$$

(25) $$F1=\frac{2}{\frac{1}{Precision}+\frac{1}{Recall}}$$

where,

*TP* = True Positives

*TN* = True Negatives

*FP* = False Positives

*FN* = False Negatives.

### 4.1. Siamese Neural Network

#### 4.1.1. Loss, Validation Loss, Accuracy and Validation Accuracy against Number of Epochs for Cell Classification

**Figure 7:**
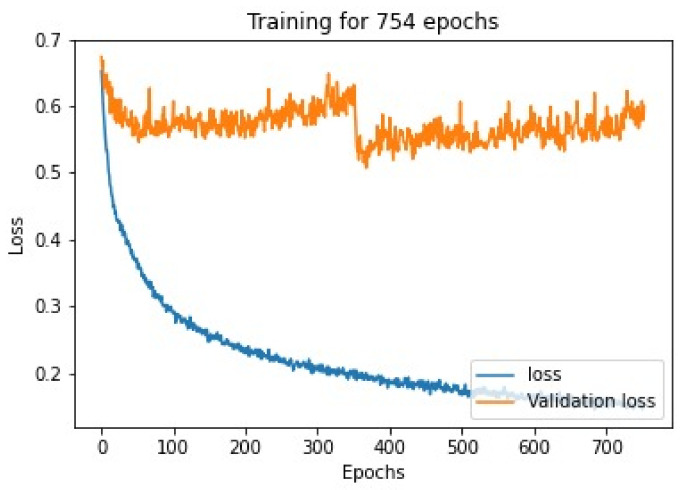
Loss v/s epoch graph for Siamese network model trained after training for 754 epochs.

The Siamese neural network model was trained on a bone marrow cell class classification image dataset, while training the loss on training dataset validation loss on validation dataset was noted, as shown in Figure 7, in every epoch, and thus the graph is plotted according to the values.

**Figure 8:**
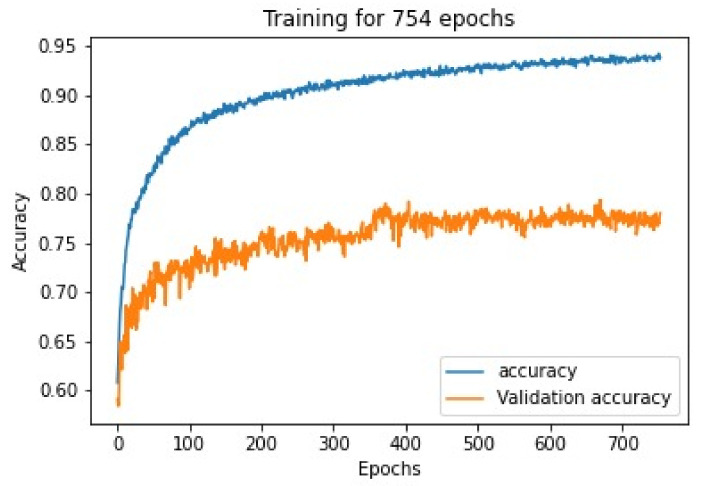
Accuracy v/s epoch graph for Siamese network model trained after training for 754 epochs.

The Siamese neural network model was trained on a bone marrow cell class classification image dataset, while training the accuracy on training dataset validation accuracy on validation dataset was noted as shown in Figure 8 in every epoch, and thus the graph is plotted according to the values.

#### 4.1.2. Similarity between Class Images

**Figure 9:**
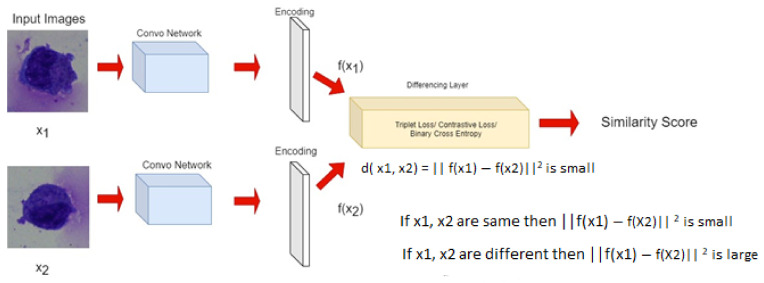
Block diagram illustrating end to end traversal in model right from the input of paired images.

Here to check the similarity between the images belonging to the same or different class, the parallel inputs A and B are created and the output, which is the distance based on loss function, is depicted as similarity percentage which depicts the confidence of images belonging to the same or different class of cells, as presented in Figure 9.

**Figure 10:**
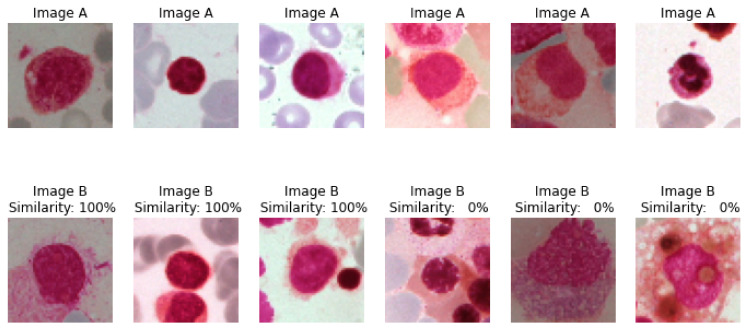
Output of model with percentage confidence in similarity for given pairs of anchor and test input images.

In the below-given Figure 10, the first three images in both the rows belongs to the same class, hence the similarity between them is 100%. The last three images in both rows belong to different classes of cells, and hence the similarity between A and B is 0%.

#### 4.1.3. Actual and Predicted Class Images

**Figure 11:**
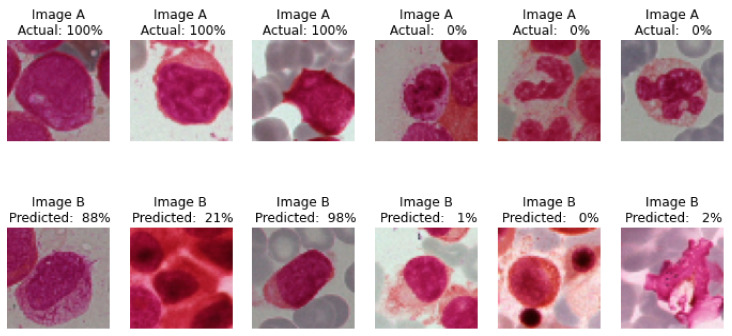
Confidence percentage of test images by Siamese model whether the input paired BM smear image belong to same class or not.

After the final training of model with certain epochs the actual and predicted images of cells are given below represented as Figure 11.

Actual 100% signifies the images A and B belongs to same class and the prediction is performed by the model which is 88%, 21% and 98%, respectively, for the same class.

Whereas Actual 0% signifies the images, A and B belong to different classes, and the prediction is performed by the model, which is 1%, 0% and 2%, respectively, for different classes.

#### 4.1.4. Confusion Matrix

**Figure 12:**
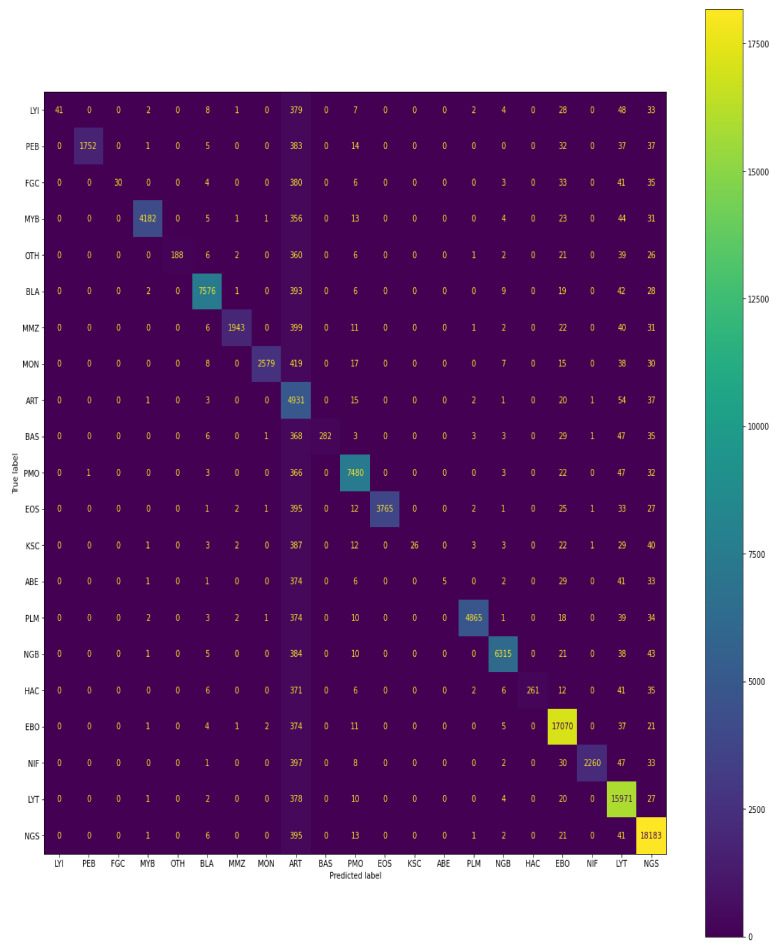
Confusion matrix of predictions generated by Siamese model on training data.

Below, in the given confusion matrix, is the visualisation of the model performance which was trained on bone marrow cell images to classify the cells based on the class of the training dataset. The visual representation is shown in Figure 12.

A total of 109,670 images were taken in the train set, out of which 41 belonged to class LYI, 1753 of PEB, 30 FGC class images, 4196 of MYB, 188 of OTH, 7662 of BLA, 1955 of MMZ, 2585 of MON, 12,563 of ART, 282 of BAS, 7676 of PMO, 3765 of EOS, 26 of KSC, 5 of ABE, 4882 of PLM, 6379 of NGB, 261 of HAC, 17,532 of EBO, 2264 of NIF, 16,794 of LYT, and 18,831 of NGS.

According to the confusion matrix shown, the model correctly classifies 41 images out of 41, 1752 images out of 1753, 30 images out of 30, 4182 images out of 4196, 188 images out of 188, 7576 images out of 7662, 1943 images out of 1955, 2579 images out of 2585, 4931 images out of 12,563, 282 images out of 282, 7480 images out of 7676, 3765 images out of 3765, 26 images out of 26, 5 images out of 5, 4865 images out of 4882, 6315 images out of 6379, 261 images out of 261, 17,070 images out of 17,532, 2260 images out of 2264, 15,971 images out of 16,794, and 18,183 images out of 18,831 for each class, respectively.

**Figure 13:**
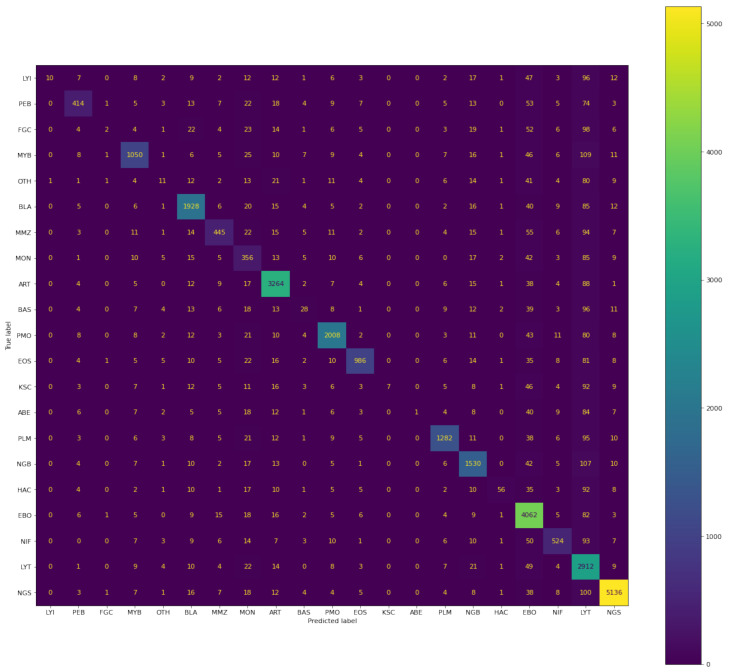
Confusion matrix of predictions generated by Siamese model on test data.

Similarly, below the confusion matrix is the visualisation of the model performance which was trained on bone marrow cells images to classify the cells based on class on the validation dataset. The visual representation is shown in Figure 13.

A total of 30,837 images were taken in the validation set, out of which 11 belonged to class LYI, 493 of PEB, 8 FGC class images, 1180 of MYB, 52 of OTH, 2155 of BLA, 549 of MMZ, 727 of MON, 3533 of ART, 79 of BAS, 2158 of PMO, 1058 of EOS, 7 of KSC, 1 of ABE, 1373 of PLM, 1794 of NGB, 73 of HAC, 4931 of EBO, 636 of NIF, 4723 of LYT, and 5296 of NGS.

According to the confusion matrix shown, the model correctly classifies 10 images out of 11, 414 images out of 493, 2 images out of 8, 1050 images out of 1180, 11 images out of 52, 1928 images out of 2155, 445 images out of 549, 356 images out of 727, 3264 images out of 3533, 28 images out of 79, 2008 images out of 2158, 986 images out of 1058, 7 images out of 7, 1 image out of 1, 1282 images out of 1373, 1530 images out of 1794, 56 images out of 73, 4062 images out of 4931, 524 images out of 636, 2912 images out of 4723, and 5136 images out of 5296 for each class, respectively.

#### 4.1.5. Evaluation Metrics

The evaluation metrics here, as shown in Table 3 and Table 4, are constructed on the precision, recall, f1 score and support values resulted from the implemented strategy for different cells’ class classification on the training and validation set. It can be observed that the weighted average recall score for training and validation of the Siamese network was 92% and 91%, respectively. As we know, recall score is a good indicator of the classification of false negatives and gives details about missed diagnostics. In that sense, a weighted average recall score of 92% for training and 91% for validation of Siamese network indicates that the model performs well in detecting anomalous cases.

#### 4.1.6. Comparison among Various Models Used

**Figure 14:**
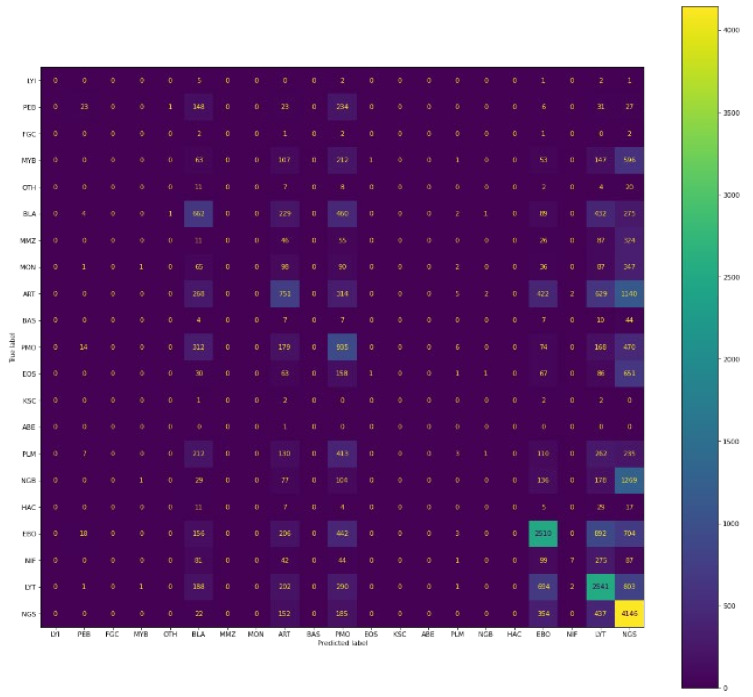
Confusion matrix of predictions generated by CNN-XGBoost.

**Figure 15:**
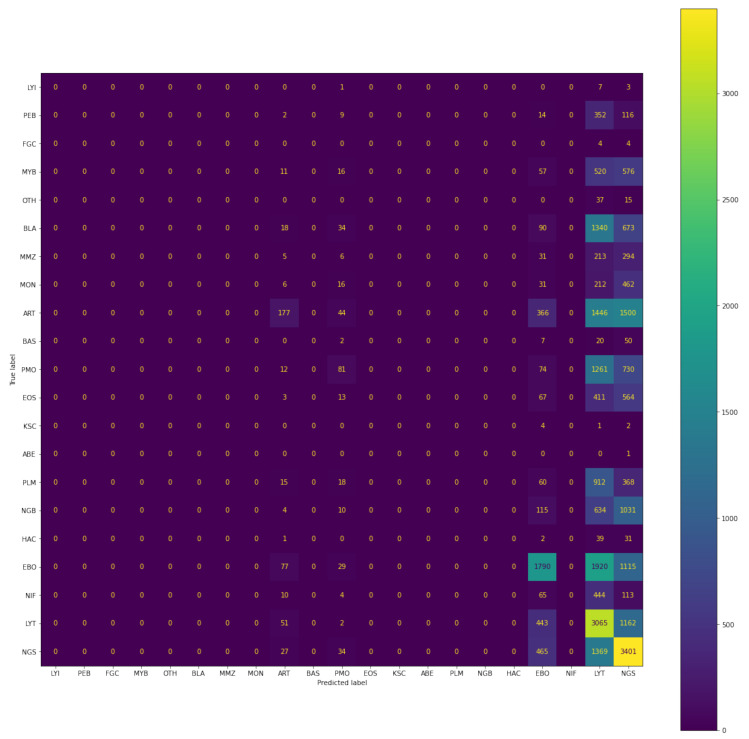
Confusion matrix of predictions made by CNN-SVM.

**Figure 16:**
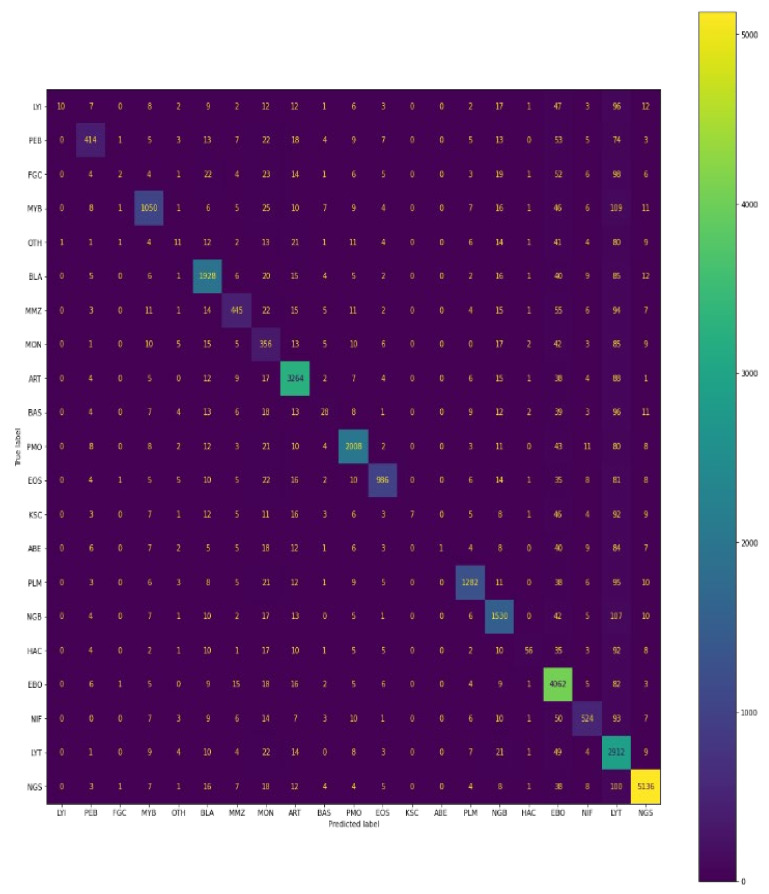
Confusion matrix of predictions generated by Siamese model.

Here, the comparison table shown in Table 5 between different models mentioned in this research is presented taking in the metric as an F1 score and the plotting of the confusion matrix represented in Figure 14, Figure 15 and Figure 16. Since F1=$\frac{2}{\frac{1}{Precision}+\frac{1}{Recall}}$, It will take into account both precision and recall. The comparison given is for validation dataset containing 30,837 images accounting for 21 classes of bone marrow cells.

#### 4.1.7. Confusion Matrix

As we can analyse from both the F1-score, accuracy, recall and as well as confusion matrix, the best performing model is Siamese neural network based on the validation set.

## 5. Discussion and Future Scope

In this paper, we present Siamese network to classify BM into 21 different classes. Substantial evaluation on 30,837 valid images has shown dominance and generalisation in predicting the labelled class with high accuracy. The model used unified Siamese architecture and CNN to classify the images. It is distinct from the existing classification models ResNeXt-50 and XGG-Boost in the sense that it does not rely on feature extraction from single image itself, rather it focuses more on similarity and dissimilarity among images between the same class and different class labels, respectively. The final accuracy on the Siamese neural network model resulted in 91% training and 84% validation accuracy. In future research, the major focus of interest will be investigation upon YOLO and Grad-CAM algorithms to focus specifically on region of interest in a single cell image, in order to reduce major dependency on convolutional unit to generate encoding for the image. In addition, we will explore various image augmentation algorithms to diminish the effect on accuracy due to class imbalance.

## Figures and Tables

**Table 1:** A summary of the findings from other papers.

Reference	Algorithm/Model Used	Performance Metric	Number of Bone Marrow Classes/Labels	Remark
7	DC-GAN + Resnet	Accuracy	4 (eosinophils, lymphocytes, monocytes and neutrophils)	91.70%
8	CNN + VGG16	Accuracy	6 (neutrophils, eosinophils, basophils, monocytes, lymphocytes (T and B cells))	94%
9	ROI Processing+ DL	Recall	16	84.20%
10	RCNN	Recall	85 images	92.68%
11	Syn-ADHA	Precision	16	87.12%
18	XGBoost	Accuracy	790,470 images	88%
10	Unet	Recall	85 images	73.17%
20	CNN + SVM	Accuracy	16	97.80%

**Table 2:** Description of 21 different class labels in the dataset.

S. No.	Class Label Acronym	Class Label Description
1	ABE	Abnormal eosinophil
2	ART	Artefact
3	BAS	Basophil
4	BLA	Blast
5	EBO	Erythroblast
6	EOS	Eosinophil
7	FGC	Faggott cell
8	HAC	Hairy cell
9	KSC	Smudge cell
10	LYI	Immature lymphocyte
11	LYT	Lymphocyte
12	MMZ	Metamyelocyte
13	MON	Monocyte
14	MYB	Myelocyte
15	NGB	Band neutrophil
16	NGS	Segmented neutrophil
17	NIF	Not identifiable
18	PEB	Proerythroblast
19	PLM	Plasma cell
20	PMO	Promyelocyte
21	OTH	Other cell

**Table 3:** Evaluation metrics score for different cell classes on train data.

Cell Class	Precision	Recall	F1-Score	Support
LYI	1.00	0.67	0.81	553
PEB	1.00	0.77	0.87	2261
FGC	1.00	0.56	0.71	532
MYB	1.00	0.90	0.94	4660
OTH	1.00	0.69	0.81	651
BLA	0.99	0.94	0.96	8076
MMZ	0.99	0.79	0.88	2455
MON	1.00	0.83	0.91	3113
ART	0.39	0.97	0.56	5065
BAS	1.00	0.36	0.53	778
PMO	0.97	0.94	0.96	7954
EOS	1.00	0.88	0.94	4265
KSC	1.00	0.75	0.86	529
ABE	1.00	0.61	0.02	492
PLM	1.00	0.91	0.95	5349
NGB	0.99	0.93	0.96	6817
HAC	1.00	0.35	0.52	740
EBO	0.97	0.97	0.97	17,526
NIF	1.00	0.81	0.90	2778
LYT	0.95	0.97	0.96	16,413
NGC	0.97	0.97	0.97	18,663
Accuracy			0.91	109,670
Weighted average	0.95	0.92	0.93	109,670

**Table 4:** Evaluation metrics score for different cell classes on validation data.

Cell Class	Precision	Recall	F1-Score	Support
LYI	0.91	0.74	0.81	250
PEB	0.84	0.63	0.72	656
FGC	0.53	0.67	0.59	271
MYB	0.89	0.79	0.84	1322
OTH	0.21	0.65	0.31	237
BLA	0.89	0.89	0.89	2157
MMZ	0.81	0.63	0.71	711
MON	0.49	0.61	0.54	584
ART	0.92	0.94	0.93	3477
BAS	0.35	0.34	0.35	274
PMO	0.93	0.90	0.91	2234
EOS	0.93	0.81	0.87	1219
KSC	1.00	0.63	0.77	239
ABE	1.00	0.54	0.70	218
PLM	0.93	0.85	0.89	1515
NGB	0.85	0.87	0.86	1760
HAC	0.77	0.61	0.33	262
EBO	0.82	0.96	0.88	4249
NIF	0.82	0.70	0.76	751
LYT	0.62	0.95	0.75	3078
NGC	0.97	0.96	0.96	5373
Accuracy			0.84	30,837
Weighted average	0.93	0.91	0.91	30,837

**Table 5:** Comparison of different approaches used in this study based on F1-Score.

Class	Siamese	CNN-SVM	CNN-XGBoost
LYI	0.81	0.00	0.00
PEB	0.72	0.00	0.08
FGC	0.59	0.00	0.00
MYB	0.84	0.00	0.00
OTH	0.31	0.00	0.00
BLA	0.89	0.00	0.30
MMZ	0.71	0.00	0.00
MON	0.54	0.00	0.00
ART	0.93	0.09	0.26
BAS	0.35	0.00	0.00
PMO	0.91	0.07	0.31
EOS	0.87	0.00	0.00
KSC	0.77	0.00	0.00
ABE	0.70	0.00	0.00
PLM	0.89	0.00	0.00
NGB	0.86	0.00	0.00
HAC	0.33	0.00	0.00
EBO	0.88	0.42	0.52
NIF	0.76	0.00	0.02
LYT	0.75	0.32	0.46
NGC	0.96	0.39	0.50
Accuracy	0.84	0.28	0.38
Weighted average	0.91	0.20	0.31

## Data Availability

The data used to support the findings are available on request. Moreover, the Bone Marrow Cell Classification dataset can be downloaded from Kaggle.
